# Identification of homologs in insignificant blast hits by exploiting extrinsic gene properties

**DOI:** 10.1186/1471-2105-8-356

**Published:** 2007-09-21

**Authors:** Jos Boekhorst, Berend Snel

**Affiliations:** 1 Bioinformatics, Department of Biology, Faculty of Science, Utrecht University, Padualaan 8, 3584 CH, The Netherlands; 2 Academic Biomedical Centre, Utrecht University, Yalelaan 1, 3584 CL Utrecht, The Netherlands

## Abstract

**Background:**

Homology is a key concept in both evolutionary biology and genomics. Detection of homology is crucial in fields like the functional annotation of protein sequences and the identification of taxon specific genes. Basic homology searches are still frequently performed by pairwise search methods such as BLAST. Vast improvements have been made in the identification of homologous proteins by using more advanced methods that use sequence profiles. However additional improvement could be made by exploiting sources of genomic information other than the primary sequence or tertiary structure.

**Results:**

We test the hypothesis that extrinsic gene properties gene length and gene order can be of help in differentiating spurious sequence similarity from homology in the gray zone. Sharing gene order and similarity in size dramatically increase the chance of a query-hit pair being homologous: gray zone query-hit pairs of similar size and with conserved gene order are homologous in 99% of all cases, while for query-hit pairs without gene order conservation and with different sizes this is only 55%.

**Conclusion:**

We have shown that using gene length and gene order drastically improves the detection of homologs within the BLAST gray zone. Our findings suggest that the use of such extrinsic gene properties can also improve the performance of homology detection by more advanced methods, and our study thereby underscores the importance of true data integration for fully exploiting genomic information.

## Background

Homology is a key concept in both evolutionary biology and genomics. Homology designates a relationship of common descent between entities. In genomics, homologs are genes or genomics regions sharing a common origin, related through speciation, duplication or a combination of both. Orthology is a specific case of homology, in which genes in different species evolved from a common ancestral gene through speciation [1]. The identification of homologous proteins is an important step in predicting the function of proteins that have not been studied experimentally and is crucial in comparative genomics studies. Identification of taxon-specific genes and estimation of the rate of gene genesis all rely on the detection of orthology (and thus homology). Rapid sequence divergence can obscure the real evolutionary relationship between genes [2], a scenario that could result in an overestimation of the contribution of the emergence of taxon-specific genes.

Algorithms like BLAST detect sequence similarity [3]. Many insignificant (by e-value) BLAST hits are nevertheless homologs. Increased sensitivity can be obtained by using more sophisticated methods, such as more accurate search algorithms like PSI-BLAST [4] and hidden Markov models (HMMs) [5]. These methods have allowed for large improvements in the identification of homologous proteins compared to BLAST, yet detection of homology might also be improved by using information not derived from the primary sequence of proteins. From here on such information is referred to as label information. We test the hypothesis that label information can make an important contribution in the detection of homology.

In order to estimate the significance of label information, we need both a method for the initial detection of sequence similarity as well as a golden standard against which we can measure the improvements made by the use of label information. The use of BLAST provides us with a commonly used method for the detections of homology, while at the same time leaving room for improvement, allowing us to use methods like HMMs as a golden standard to benchmark gray zone BLAST hits.

The first label we investigate in this study is gene order. In the absence of changes in gene order (i.e. if no rearrangement, deletion or duplication of genes were to occur), even genes with no detectable sequence but sharing gene order would be homologous. Gene order has been proven to be a useful tool in the identification of homologs with low sequence similarity, as illustrated for non-coding RNA genes [6]. We test the hypothesis that proteins sharing gene order and identified in a BLAST search as being weakly similar in sequence (gray zone hits) are more likely to be homologous than proteins with the same sequence similarity that do not share gene order. The reasoning for the second label we study, protein length, is similar. Unless gene fusion or fission events have taken place, gene sharing a common ancestor and retaining the same fold should be similar in length. A substantial difference in length between two proteins suggests a difference in three dimensional structure, in turn suggesting a different evolutionary origin; two proteins with similar sizes are more likely to be homologs than two proteins with a relatively large difference in size.

Here we test whether label information can play an important role in the identification of homologs in the gray zone. We use the prokaryotic protein sequences from the COG database [7] as dataset in an all-against-all BLAST, and the PFAM database as a golden standard. We discuss the implications of our findings for the detection of sequence homology with more advanced methods, the identification of taxon specific genes, and the importance of true data integration.

## Results

### The value of label information

Here we test the expectation that label information can play an important role in the identification of homologs in the gray zone by comparing the results of an all-against-all BLAST search of the proteins from the COG database to data from the PFAM database. The gray-zone of BLAST is not systematically defined; normally, it represents a range of values starting directly above some e-value threshold up to BLAST's default maximum e-value of 10. We here refrain from directly designating a grey zone and instead vary the e-value threshold. For values which are normally considered grey-zone, we then observe as expected that a substantial portion of hits are not homologous according to PFAM: 65% of the BLAST hits with an e-value above 1e-03 but below 10 are homologous according to PFAM clans, and only 43% of the hits are homologous at e-values between 1 and 10. In contrast, over 99% of the hits below a threshold of 1e-03 are homologous according to PFAM clans, also as expected.

**Figure 1:**
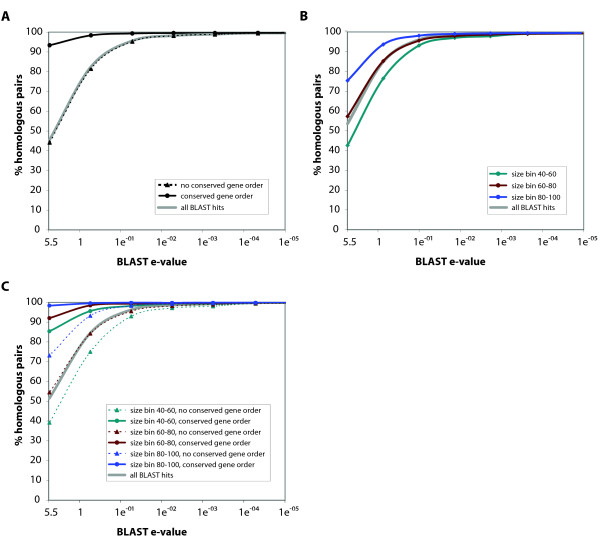
**A-C: Homologous query-hit pairs according to PFAM clans**. The pairs were binned by e-values; each tick mark in the graph corresponds to the middle of a score bin. In figures 1B and C, pairs were also binned by relative size difference (the difference is defined as the relative size of the smallest protein of a query-hit pair compared the largest in %)

If we now take into account gene order, we observe that the percentage of homologous hit pairs is increased dramatically when query and target share gene order (Figure 1A); for example, 98% of the gray-zone query-hit pairs with conserved gene order are homologous, compared to 69% of the pairs without conserved gene order. In other words, we go from a fraction of false positives of 0.31 to only 0.02, a 15-fold reduction in the fraction of false positives. Gene length shows similar behaviour (Figure 1B): query-hit pairs of similar size (the length of the smallest protein at least 80% of that of the largest protein) are homologous in 87% of all cases, while for pairs with a large difference in size (40%–60%) this is 56%. We thus go from a fraction of false positives of 0.44 to 0.13, a 3-fold reduction in the fraction of false positives.

Combining gene order and gene size (Figure 1C) further increases the differentiation between homologous query-hit pairs and spurious BLAST hits. Query-hit pairs with gene order conservation and with similar sizes (80%–100%) are homologous in over 99% of all cases, while for query-hit pairs of different sizes (40%–60%) and without conserved gene order this is 55% (Figure 1C). Expressed as a reduction in the fraction of false positives, this improvement is quite high: from 0.45 to 0.01 is a 45 fold reduction. Note that because of the relatively small number of genes with conserved gene order (more on this below) the inclusion of gene order does not have a big impact on the precision when looking at the fraction of homologous pairs with a large size difference (56% for all pairs, 55% for the pairs without conserved gene order).

### Comparing the contributions of conserved gene order and size similarity

**Figure 2:**
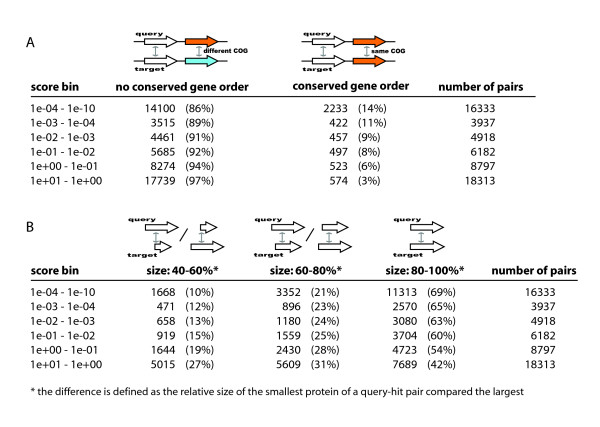
**A and 2B: Relative number of hit pairs per context, score and relative size**. To normalize for the wide variation in number of hits per query protein, a query-hit pair contributes 1/(total number of hits of query) to the relevant bin. Figure 2A shows the number of pairs with and without gene order conservation, Figure 2B the number of pairs per size bin.

The data presented above shows that both having conserved gene order and being of similar size increases the chance of gray-zone BLAST hits being homologous. Size and gene order do not offer the same increase in confidence; having conserved gene order means an increase in the percentage of homologous query-hit pairs from 42% to 93% for pairs scoring between 1 and 10, while being of similar size means an increase from 32% to 70% (Figures 1A and 1B). On the other hand, only 3% of the query-hit pairs with an e-value between 1 and 10 have conserved gene order, while 42% of the pairs in this score bin belong to the size bin of proteins most similar in length (Figures 2A and 2B). In summary, the proportion of query-hit pairs with conserved gene order is smaller, but when encountered conserved gene order is more informative than being of similar size.

### Estimating the number of additional homologs that can be detected

**Figure 3:**
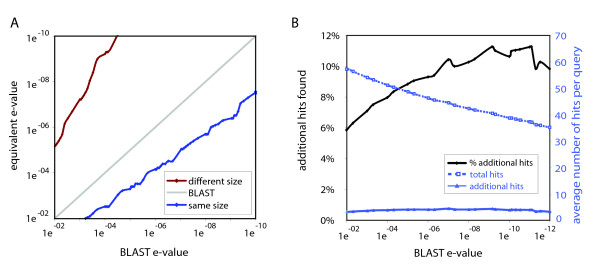
**A and 3B: Equivalent e-values and additional BLAST hits**. Figure 3A shows equivalent e-values for the label gene size. We defined the equivalent e-value of a specific label as the e-value threshold that gives the same positive predictive value as a BLAST search not using label information (a more detailed description is given in the main text). Figure 3B shows the average number of homologs per query when equivalent e-values are used as thresholds in cases where genes of a query-hit pair share gene order or are of similar size.

In order to estimate the number of additional BLAST hits that the use of label information yields, we employ a heuristic framework to determine the equivalent e-value for a search with and without label information. The equivalent e-value is determined approximately as follows: For each BLAST e-value threshold, hits scoring below that threshold have a certain chance of being homologous (as represented by the positive predictive value, see methods). The same positive predictive value is observed at higher e-values for blast hits with similar length compared to normal blast hits (Figure 3A). We call these two different e-value thresholds that give the same performance equivalent, and they allow us to translate the e-value of normal BLAST hit to the equivalent e-value of a BLAST hit with similar length. For example, the positive predictive value of a BLAST search with an e-value threshold of 1e-03 is 0.998 (in other words, approximately 0.2% of the hits scoring below 1e-03 are not homologous). When we now take BLAST hits with the same gene size, this PPV is attained at e-value 1.4e-02. Using this translated e-value we can show how many more blast hits one can find using label information while maintaining the same performance. Figure 3B shows the number of BLAST hits that can be recovered using equivalent e-values and the labels gene order and gene size. For e-value thresholds from 1e-02 to 1e-12, between 6% and 12% extra hits are found when we use label information.

### Examples

**Figure 4:**
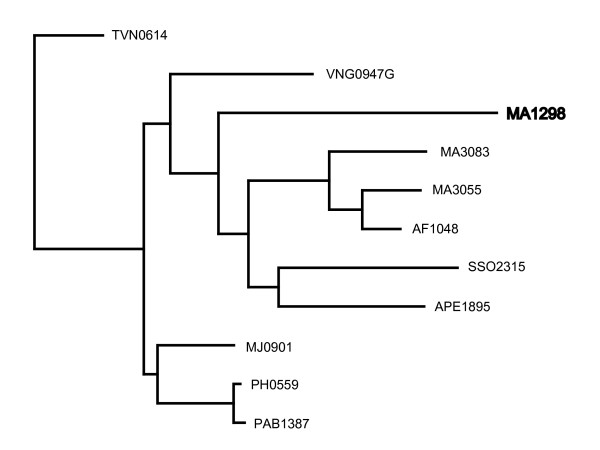
**Phylogenetic tree of the proteins belonging to COG1955 and the *Methanosarcina acetivorans *protein MA1298**. For visualization purposes, we show a subset of the COG1487 proteins (the total number of proteins belonging to COG1487 is 84). Proteins starting with MT belong to *M. tuberculosis*. The tree was visualised with TreeView [26].

The protein MA1298 from *Methanosarcina acetivorans *has no significant BLAST hits (e-value < 1e-02). MA1298 is not part of any orthologous groups of the COG database, while one of its BLAST hits, the protein APE1895 from *Aeropyrum pernix*, belongs to COG1955, which contains proteins involved in flagella biosynthesis. Since the two proteins share gene order (both have a neighbour belonging to COG0630, a group containing transport proteins involved in flagella biosynthesis) and are similar in size (APE1895 is 85% of the size of MA1298), we expect MT0685 to be homologous to the proteins from this COG. We confirmed this using PSI-BLAST: after 3 iterations with APE1895 as query, MA1298 is hit with an e-value of 7e-49. A phylogenetic tree of MA1298 and the proteins comprising COG1955 shows MA1298 to be orthologous to the proteins belonging to COG1955 (Figure 4). Thus because of conserved gene order and similarity in size we not only recognize the BLAST hits of MT0685 as homologs despite their high e-value, we are also able to improve the COG database. This observation does not diminish the usefulness of COG, which is created automatically and provides a comprehensive definition of orthologous groups, while the example given above is the result of manual selection and analysis. Rather, this example illustrates that gene order and gene size can be used in parallel to other methods such as BLAST.

A well-known problem in the detection of homology using sequence profiles is the threshold effect: creating a sequence profile and setting a threshold that excludes all non-homologous sequences while at the same still recognizing all homologs can be very difficult. To illustrate the value of gene order and gene length in such scenarios, we mined our data set for gray-zone query-hit pairs of similar size and with conserved gene order, but where only one of the two proteins is hit by a PFAM model. These pairs were left out of our original analysis, as for these pairs we can not reliably automatically establish if they are homologous according to our golden standard. The protein SA0534 from *Staphylococcus aureus *hits the protein AGl121 from *Agrobacterium tumefacie *(e-value: 0.6). SA0534 is member of protein family PF00108 (Thiolase), while AGl121 is not a member of any protein family. However, conservation of gene order and similarity in length suggest that the proteins are homologous. Again we could confirm this using PSI-BLAST: after 3 iterations SA0534 hits AGl121 with an e-value of 2e-52. A multiple sequence alignment of AGl121 with the members of PF00108 shows the cysteine residue experimentally proven to be essential for thiolase activity [8] to be conserved.

## Discussion

### The value of label information

We have shown that query-hit pairs of similar size and sharing gene order are much more likely to be homologous than pairs less similar in size and not sharing gene order. Obviously, this effect is most dramatic for gray-zone hits: query-hit pairs without gene order conservation and with different sizes (the length of the smallest protein between 40% and 60% of that of the largest protein) are homologous in only 55% of all cases, while for query-hit pairs of similar size (80%–100%) and conserved gene order this is 99% (Figure 1). For an e-value threshold between 1e-02 and 1e-12, the use of the labels gene size and gene order provides an average increase in the number of hits ranging from 6% to 12% (Figure 3B). Using label information can mean the difference between ignoring a gray zone BLAST hit as spurious and recognizing a distant homolog.

The small fraction of gray zone query-hit pairs with conserved gene order compared to gray zone hits of similar size (Table 1B,C) means that conserved gene order has less impact on the overall picture. In addition, an a priori advantage of length as a label over conserved gene context is that the use of conserved gene order is in practice limited to prokaryotes, because in eukaryotes gene order evolves relatively fast compared to protein sequences [9].

Note that the use of gene order in this context is not so obvious, because it does not equal the use of conserved gene order for function prediction, such as it is applied in genomic context methods [10,11]. In fact conserved gene order would not even have been detected if only blast were used (as is often the case), as one of the genes in a pair of neighbouring genes does not have a significant blast hit in the other species. The use of gene order as a label is here thus the reverse approach of the genomic context method: we suppose the conservation of gene order as a clue.

### Fusion of proteins

As the COG database offers an orthology prediction we could use for the detection of conserved gene order, and because of the summary of an all-against-all BLAST provided with the database, we used the proteins from COG as a starting point. A potential problem with this dataset is the presence of fusion proteins in the COG database as separate entries [7]. We investigated the effect of this lack of fusion proteins on size similarity as a relevant label by concatenating the relevant protein entries our analysis with this alternative set. The results show proteins size to be a relevant label for recognizing gray zone homologs also in datasets containing fusion proteins.

### Methods other than BLAST

BLAST is applied extensively, yet in many scenarios more advanced methods are more suitable. The observation that label information can be of great value in interpreting the results of a BLAST search could be extrapolated to more advanced methods. A somewhat trivial example of this is given in the results section, where we show via label information that two sequences likely belong to the same PFAM family, even though only one of them is hit by the PFAM model above the inclusion bitscore threshold. In related work [12], the label taxonomy is shown to enhance the detection of PFAM domains.

In the field of distant homology detection, the challenge has become the detection of homologous domains (e.g. profile vs. profile alignment and fold prediction [13,14]). The applicability of the labels for this field might be difficult, but we think the label protein length could perhaps be fairly directly translated to the length of conserved domains. It is very unlikely to find such a direct analogy for conserved gene order, but more tenuous analogs could be found among the co-occurrence [15] and relative order of domains in a protein, or to the more general concept of functional links, such as co-expression or participating in the same pathway (thus implicitly assuming the duplication of functional modules). Note also that investigating the value of label information for more advanced methods such as PSI-BLAST provides a practical challenge in the sense that it requires an even more reliable method or database to provide a golden standard.

### Implications

One of the scenarios in which improved homology detection and thus label information can have a big impact is the estimation of percentage of so-called novel genes: genes for which no homologs could be identified in other organisms. BLAST is often the method of choice for the detection of genes specific to a species or taxon [16-18], as BLAST can make use of all available sequences, while many methods based on sequence models like PFAM and SCOP [19,20] do not cover the full spectrum of available sequence data. Almost two-thirds of the proteins encoded by the genome of *Plasmodium falciparum *did not have sufficient similarity to proteins in other organisms to justify provision of functional assignment, which led the authors to state that two-thirds of the proteins appear to be unique to the organism [21]. The use of label information would allow a more accurate identification of proteins that in fact do have homologs in the set of proteins with only gray zone BLAST hits, in turn leading to a decrease in the fraction of unique proteins.

There has been a call for so-called true data integration [22]: tools and databases should not only combine different types of biological data in the sense that they provide access to different types of analysis or data sources through a single interface, they should in fact combine different types of data to come to a single answer taking into account as much of the available data as possible. This would, in the case of BLAST and the labels gene size and gene order, be a tool that combines a similarity search, gene size, and information on genome organization into a statistical framework that produces a single score representing the likelihood of a hit being homologous to the query. The use of other labels like co-expression and phylogeny requires an extensive integration of databases and tools, yet as we have shown the gain is potentially substantial.

## Methods

### Sequence information and analysis

Protein sequences were taken from the COG database [7]. As one of the two labels we are interested in is gene order conservation, and gene order evolves more slowly in bacteria [9], we excluded the three genomes of eukaryotes, leaving us with 63 bacterial genomes with a total of 192987 protein sequence. Gene order information was taken from Genbank [23] after mapping COG identifiers to Genbank gene identifiers. Gene order was considered to be conserved when two genes have one or more neighbours belonging to the same COG. We used BLAST [4] for the detection of sequence similarity. Where possible we took advantage of the pre-calculated BLAST data provided by the COG database. Information on protein families was taken from version 20.0 of the PFAM database [19]. In the cases where an exact match of a protein in our data set could not be identified in the PFAM database, we assigned protein families using HMMer [5] and the models provided by PFAM; in all other cases we used the pre-calculated results distributed with the database. Multiple sequence alignments were made with MUSCLE [24], phylogenetic trees were constructed using PhyML [25].

### Measuring the value of label information

We measured the value of the label information in identifying homologous by binning all query-hit pairs from the all-against-all BLAST provided with the COG database by e-value and similarity in protein length (defined as the relative size of the smallest protein of a query-hit pair compared the largest in %). For each bin we counted the homologous pairs sharing gene order, the homologous pairs not sharing gene order, the not-homologous pairs sharing gene order and the not-homologous pairs not sharing gene order. Numbers of hits were normalized by dividing by the number of hits in that category of a query protein by the total number of hits of that query in order to prevent large protein families (which have many very similar hits) from skewing the results. The chance that the two proteins of a query-hit pair in a specific score bin and with specific label properties are homologous (also called the positive predictive value (PPV) or precision) was calculated by dividing the number of homologous pairs (true positives) by sum of the number of homologous pairs and the number of non-homologous pairs (true positives + false positives).

Our methodology requires both a golden standard for defining homologous proteins as well as a standard for defining non-homologous proteins. Pairs were considered homologous when the proteins belong to the same protein family (as defined by the PFAM database) or to protein families belonging to the same clan [19], while pairs were considered to be non-homologous when they were part of protein families not belonging to the same clan. The number of false negatives was reduced by excluding pairs belonging to different PFAM clans while at the same time belonging to the same orthologous group of the COG database. Query-hit pairs formed by one or two proteins not belonging to any protein family were left out of the analysis; this keeps protein pairs that are in fact homologous, but that are not covered by any of the models in the PFAM database, as well as proteins that are hit by a PFAM model but fall just below the gathering threshold established by PFAM, from being counted as non-homologous.

## Competing interests

The author(s) declares that there are no competing interests.

## Authors' contributions

BS conceived the study, participated in its design and coordination and contributed to the manuscript. JB performed the analysis and drafted the manuscript. Both authors read and approved the final manuscript.
